# Clinical epidemiology, determinants, and outcomes of viral encephalitis in Ghana; a cross-sectional study

**DOI:** 10.1371/journal.pone.0297277

**Published:** 2024-02-12

**Authors:** Richmond Yeboah, Richmond Gorman, Henry Kyeremateng Acheampong, Emmanuella Nyarko-Afriyie, Sherihane Aryeetey, Henrietta Dede Tetteh, Michael Owusu, Eric Smart Yeboah, Titus Adade, Joseph Bonney, Yaw Ampem Amoako, Philip El-Duah, Kwasi Obiri-Danso, Christian Drosten, Richard Odame Phillips, Augustina Angelina Sylverken

**Affiliations:** 1 Kumasi Centre for Collaborative Research in Tropical Medicine, Kumasi, Ghana; 2 Department of Theoretical and Applied Biology, Kwame Nkrumah University of Science and Technology (KNUST), Kumasi, Ghana; 3 Department of Medical Diagnostics, Kwame Nkrumah University of Science and Technology (KNUST), Kumasi, Ghana; 4 Emergency Unit, Nyaho Medical Centre, Accra, Ghana; 5 Department of Medicine, Komfo Anokye Teaching Hospital, Kumasi, Ghana; 6 Emergency Medicine Directorate, Komfo Anokye Teaching Hospital, Kumasi, Ghana; 7 Department of Medicine, School of Medicine and Dentistry, Kwame Nkrumah University of Science and Technology (KNUST), Kumasi, Ghana; 8 Institute of Virology, Charite, Universitätsmedizin Berlin, Berlin, Germany; University of Warwick, UNITED KINGDOM

## Abstract

Viral encephalitis is a rare, yet severe neurological disorder. It poses a significant public health threat due to its high morbidity and mortality. Despite the disproportionate burden of the disease in impoverished African countries, the true extent of the problem remains elusive due to the scarcity of accurate diagnostic methods. The absence of timely and effective diagnostic tools, particularly Real-time Polymerase Chain Reaction, has led to misguided treatment, and an underestimation of the disease burden in Ghana. We conducted a prospective cross-sectional study to determine the viral aetiologies of encephalitis among patients presenting to a major referral hospital in Ghana from May 2019 and August 2022. The study aimed at providing a comprehensive information on the clinical epidemiology, and outcomes of viral encephalitis in Ghana. Clinical samples were collected from patients presenting with signs and symptoms of encephalitis and tested for viral agents using real-time polymerase chain reaction. We assessed the clinical epidemiology, risk factors and outcome of individuals using descriptive and logistic regression analysis. Seventy-seven (77) patients were enrolled unto the study. The participants frequently presented with fever (85.7%), seizures (80.5%), lethargy (64.9%) and headache (50.6%). Viruses were detected in 40.3% of the study participants in either cerebrospinal fluid, rectal or oral swab samples. The most frequently detected viruses were cytomegalovirus (48.4%), enteroviruses (38.7%) and HSV (29.0%). Twenty-one (27.3%) of the patients died while on hospital admission. Gender (OR = 5.70 (1.536–1.172), *p = 0*.*01*), and negative polymerase chain reaction test results were identified as significant factors associated with death. Antiviral treatment increased the chance of survival of viral encephalitis patients by 21.8%. Our results validate the crucial role of molecular tools as essential for the rapid diagnosis of viral encephalitis, enabling effective treatment and improved patient outcomes. This study contributes valuable epidemiological and clinical insight into viral encephalitis in Ghana.

## Introduction

Encephalitis is an inflammation of the functional tissues of the brain and is characterised by an acute onset of fever, altered mental status and the onset of seizures. Although rare, encephalitis affects patients of all ages and represents an important public health challenge globally due to the high morbidity and mortality associated with the condition [1], with the greatest impact in tropical regions of the world. Globally, 3.5–7.4 cases are reported for every 100,000 individuals who present at the hospital annually and is one of the leading causes of fatality in the paediatric population in sub-Saharan Africa (SSA) [2]. The significant burden of encephalitis is evident in the economic costs of hospitalizations. In 2010, approximately $2 billion was spent in the United States of America on encephalitis hospitalisations [3].

Although not all cases of encephalitis have an infectious aetiology, infectious encephalitis accounts for most cases recorded in the tropics [4]. Viruses constitute the major aetiology for encephalitis and molecular diagnostic techniques such as Polymerase Chain Reaction (PCR) and genome sequencing are the preferred diagnostic techniques for confirmation of viral aetiology. These techniques have aided in diagnosing viral aetiologies of encephalitis in the developed world [5–7]; however, such techniques are not widely available in the African setting. In Ghana, the case is not different, consequently, the diagnosis of any form of encephalitis has largely been presumptive. Such a syndromic diagnostic approach leads to delayed diagnosis, challenges with elucidation of the aetiology (due to limited diagnostic capacity), inadequate treatment targeting, irrational use of drugs (particularly antibiotics), prolonged hospitalization, and poor disease outcomes. Increased utilization of health services, and high socio-economic costs further challenge health systems in low resource settings like Ghana. These challenges potentially increase the burden of encephalitis in endemic countries. A further challenge with encephalitis is that inadequate disease characterization by clinicians translate directly into lack of mandatory notification of the relevant public health authorities and this contributes to the dearth of data on encephalitis in the African region. This poor characterization of the true disease burden may hamper policy making and planning by public health authorities in resource poor settings.

Molecular diagnostic tools are essential for the adequate characterisation of the aetiology of encephalitis, but these tools are not widely available or are unaffordable in resource poor settings where the burden of encephalitis is highest and such tools may be most needed. Molecular diagnostics has aided in elucidating a few cases of viral encephalitis in Ghana [8–10], but these reports were focused on individuals with specific conditions. At present, there is no study that has comprehensively determined the burden, clinical epidemiology, risk factors and outcomes of viral encephalitis in Ghana. To the best of our knowledge, no such comprehensive report is available on encephalitis in West Africa or Africa as a whole. Infectious pathogens of the central nervous system in this region are only known through sporadic case reports [4].

This study therefore sought to determine the viruses associated with encephalitis among patients presenting to a major referral hospital in Ghana. We aimed to assess the clinical epidemiology, risk factors, and outcome of individuals who presented with signs and symptoms of encephalitis in a tertiary hospital setting in Ghana.

## Materials and methods

### Study design and participants

This was a prospective cross-sectional study which was undertaken among individuals presenting to the Komfo Anokye Teaching Hospital (KATH). KATH is the largest and the only tertiary hospital in the Ashanti Region located in the middle belt of Ghana and receives referrals from the other fifteen administrative regions of the country.

Recruitment was non-randomised and purposive. All consenting patients reporting to the Emergency Medicine, Internal Medicine and Child Health Directorates of KATH who presented with signs and symptoms of neurological infection between May 2019 and August 2022 were considered for the study.

#### Inclusion criteria

Patients who had experienced at least two cardinal signs of encephalitis (headaches, fever, stiffness of the neck, and impaired consciousness) within the preceding six weeks prior to hospital admission were recruited. Patients with secondary symptoms such as hallucinations, sleep disorders, vomiting, speech disorders and within 72 hours prior to hospitalisation were also included. Additionally, individuals with cardinal symptoms including a depressed or altered level of consciousness lasting 24 hours or longer, lethargy, or a personality change accompanied by at least two of the following: fever, seizure, focal neurologic impairment were also considered eligible for the study.

#### Exclusion criteria

Patients with pre-existent neurological conditions and patients whose neurological signs and symptoms were confirmed to be due to bacterial meningitis, stroke, neuro-invasive parasites, cranial traumatism, and cerebral malaria were excluded from the study.

### Sampling

After obtaining informed consent, clinicians collected samples from participants for the study measurements. Samples collected were oral and rectal swabs, as well as cerebrospinal fluid (CSF). The samples were appropriately marked, labelled, and transported to the laboratory for further analysis.

For rectal swabs, the individual was placed in the left lateral decubitus position with flexion at the knee and hip. A sterile flock-tipped swab (Copan, Brescia-Italy) was used to collect specimen from the rectal mucosa. To ensure optimal sampling, swabbing was included in all four quadrants (3, 6, 9, 12 o’clock positions) of the rectum. Rectal swabs were placed in a sterile 1.5 ml Eppendorf tube containing ribonucleic acid (RNA) preservation medium (RNAlater, ThermoFisher Scientific, USA) and sealed off for onward transport to the laboratory for processing. To avoid contamination, care was taken to ensure the swab did not touch any object or surface before and after swabbing the rectal mucosa.

Oral swabs were collected using sterile flock tipped swabs. With the participant’s mouth open, the oral mucosa was swabbed on all sides and the gum inferiorly and superiorly. Swab samples were placed in a sterile 1.5 ml Eppendorf tube containing RNA preservation medium and the lids were securely sealed. To avoid contamination, care was taken to ensure the swab did not touch any object or surface before and after swabbing the oral mucosa.

Lumbar puncture was performed for collection of CSF samples using standard procedures after the study clinician had ruled out any contraindications such as high intracranial pressure and trauma to lumbar vertebrae for the procedure. Briefly, the patients were placed in the lateral recumbent position with the neck, back, hip and limbs held in maximal flexion. Under strict asepsis and after application of local anaesthetic, a spinal needle was inserted in the subarachnoid space between the third lumbar (L3) and fourth lumbar (L4) vertebrae. As soon as CSF appeared and began to flow through the needle, a digital manometer (Centurion, USA) was placed over the hub of the needle and the opening pressure was measured and recorded. Serial 1ml aliquots of CSF were collected into sterile containers for virologic analysis at the Kumasi Centre for Collaborative Research in Tropical Medicine.

Socio-demographic details of participants such as age, sex, educational level, and occupation were collected using a structured questionnaire. Further, clinical information was abstracted from the patient folders. Information collected included exposure history, clinical symptoms and signs of encephalitis, duration of illness, treatments received, results of investigations, final diagnosis, and clinical outcome.

### Laboratory analysis

Two commercial kits; Wellcogen™ Bacterial Antigen Kit, (Oxoid, ThermoFisher Scientific, USA) and First Response Malaria Ag. (pLDH / HRP2) Combo Card Test (Premier Medical Corporation Ltd., Mumbai, India) were used to test for antigens to selected bacteria (*Streptococcus* group B, *Streptococcus pneumoniae*, *Haemophilus influenzae* type b, *Neisseria meningitidis* groups A, B, C, Y or W135 and *Escherichia coli* K1) and malaria parasites in CSF respectively, according to manufacturers’ instructions to exclude any case of bacterial meningitis or cerebral malaria.

#### Viral panel

**Fig 1:**
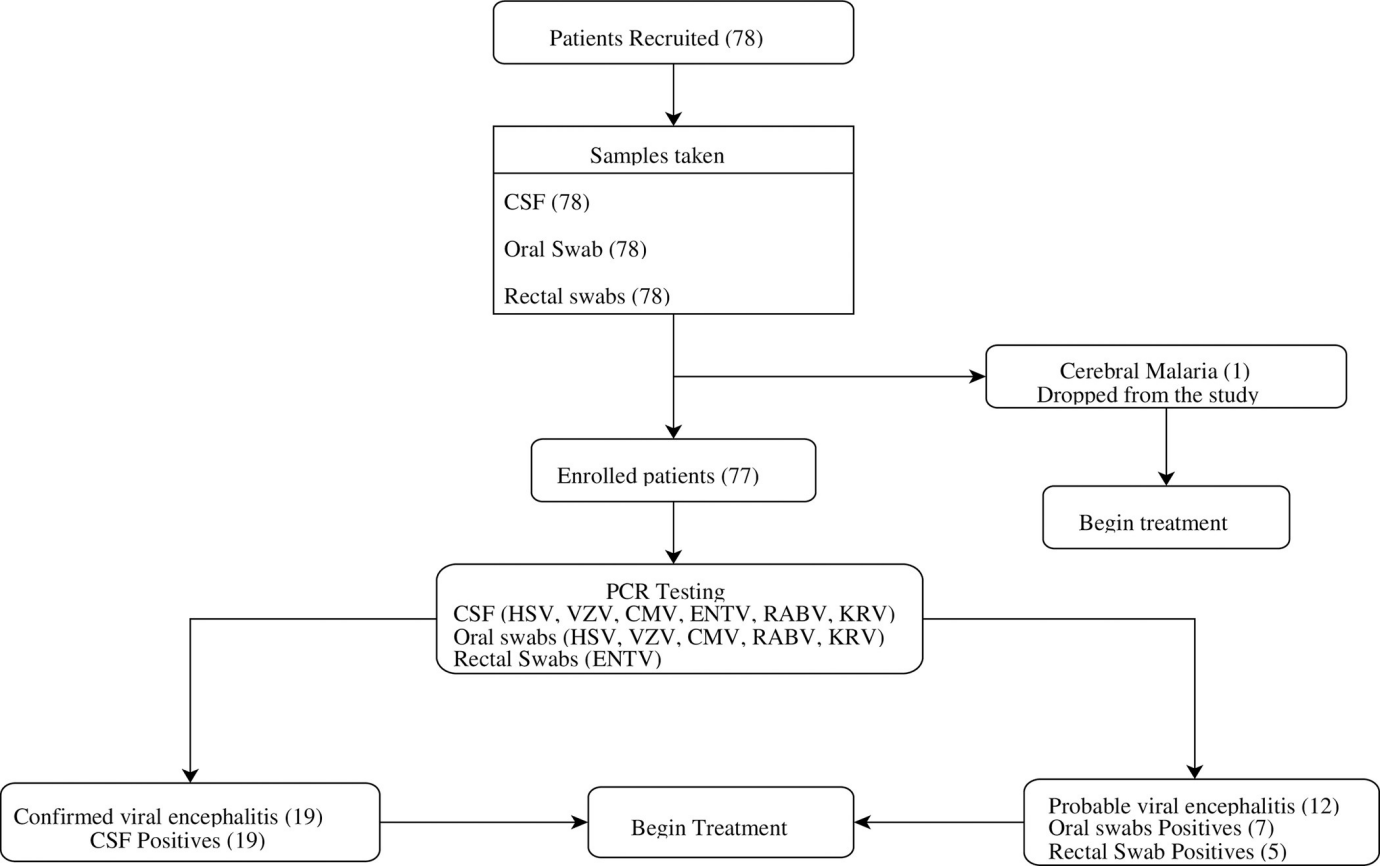
Diagnostic algorithm of viral encephalitis.

Viral testing was done by extracting viral nucleic acid from CSF, oral and rectal swab samples using the Qiagen Viral RNA mini kit (Qiagen, Hilden-Germany) according to manufacturer’s instructions. The extracted nucleic acid served as template for subsequent PCR. The nucleic acids were tested for a panel of six viruses. The panel included three RNA viruses (rabies, enteroviruses, and Kumasi Rhabdovirus) and three deoxyribonucleic acid (DNA) viruses (varicella zoster, herpes simplex and cytomegalovirus) as outlined in Table 1. The Kumasi Rhabdovirus, which has not been known to be associated with encephalitis, was tested to determine whether it played a role in causing viral encephalitis. The Invitrogen Superscript III One Step RT-PCR system with Platinum Taq DNA (ThermoFisher Scientific, USA) was used for the PCR runs using a gradient real time PCR for detection of both DNA and RNA viruses concurrently. Briefly, cycling conditions for the reaction were initial reverse transcription (for RNA viruses) at 50°C for 30 minutes, initial denaturation at 95°C for 3 minutes, followed by a denaturation step at 95°C for 15 seconds. Annealing was achieved at a gradient range of 54–63°C for 30 seconds. The denaturation and annealing steps were repeated for 45 cycles. Acquisition was done after each annealing step. Bio-Rad CFX96 C1000 thermal cycler (Bio-Rad Laboratories Inc., USA) was used for the cycling and analyses of the PCR results. Known negative and positive samples for the various targets of interest were included in the testing as negative and positive controls respectively for results validation. Any sample with cycle threshold (ct) value ≤38 was regarded as positive. Samples with clear amplifications but had ct values greater than 38 were repeated in duplicates. If all replicates amplified, they were considered positive. Viral encephalitis was confirmed for a patient if there was amplification by PCR on a CSF sample. Any amplification in either oral or rectal swab was considered probable viral encephalitis. The entire flow of the study was as presented in Fig 1. Authors had no access to information that could identify individual participants during or after data collection.

**Table 1:** Viruses tested and primer/probe sequences.

Virus	Function	Oligonucleotide sequence	Reference
**Cytomegalovirus (CMV)**	Forward	CGT TGG TGT TGT AGC AAC TGG C	[11]
	Reverse	TGT GCT CAA AGA GGT CGA GTT CC	
	Probe	FAM-CGC GAA GGT GTG GCG GCA G-BHQ1	
**Herpes simplex virus (HSV)**	Forward	HSV-S-5^’^-CAT CAC CGA CCC GGA GAG GGA C-3’	[12]
	Reverse	HSV-As- 5^’^-GGG CCA GGC GCT TGT TGG TGT A-3’	
	Probe	HSV-P-FAM-5^’^-CCG CCG AAC TGA GCA GAG ACC CGC GCT-BHQ1-3’	
**Enterovirus (ENTV)**	Forward	EQ1-5’-ACA TGG TGT GAA GAG TCT ATT GAG CT-3’	[13]
	Reverse	EQ2-5’-CCA AAG TAG TCG GTT CCG C-3’	
	Probe	EP-FAM-5^’^-TCC GGC CCC TGA ATG CGG CTA AT-BHQ1-3	
**Rabies virus (Pan Lyssa)**	Forward 1	ACGCTTAACAACCAGATCAAAGAA	[14]
	Forward 2	ACGCTTAACAACAAAATCADAGAAG	
	Reverse	CMGGGTAYTTRTAYTCATAYTGRTC	
	Probe 1	(FAM) AA+C+ACCY+C+T+ACA+A+TGGA (BHQ1)	
	Probe 2	(FAM) AA +C +ACTA +C +T +ACA +A +TGGA (BHQ1)	
**Rabies virus (RABV)**	Forward	GGTTTCCGGDGCYGTDCCTC	[15]
	Reverse	CCTAGGGGAGACYTTGCCRT	
	Probe	6FAM-CCCGTCAYATAGGGTCRGCTCARGGGC–BBQ	
**Varicella -zoster virus (VZV)**	Forward	5^’^-TGT CCT AGA GGA GGT TTT ATC TG-3’	[16]
	Reverse	5’-CAT CGT CTG TAA AGA CTT AAC CAG T-3’	
	Probe	FAM-5^’^-AAG TTC GCG GTA TAA TTG TCA GTG GCG T-BHQ1-3’	
**Kumasi rhabdovirus (KRV)**	Forward	CTGACTATCGCGACATGCTGTAC	[17]
	Reverse	TCCATTGCTCTCTGGCTCAA	
	Probe	FAM-ACACGGCGAAAGATCATGCCAAACA-(BHQ)	

### Data management and statistical analysis

Data collected by paper-based case report forms were entered into the Research Electronic Data Capture (REDCap) web application (Vanderbilt University, TN-USA) hosted by the School of Medicine and Dentistry of KNUST. All data were exported to Microsoft excel version 2019 (Microsoft Corporation, Washington, USA) and checked for inconsistencies and subjected to analyses using the R statistical software version 4.2.1 (The R Foundation, Auckland, USA). Summary frequencies and proportions were used to describe all nominal characteristics including patients’ sex and symptoms. Median, mean and inter quartile range were used to describe continuous variables depending on the distribution of the variables. Logistic regression was performed on selected variables (with p-value <0.2). For all analysis, a p-value of less than 0.05 was considered as statistically significant.

### Ethics

The Committee for Human Research, Publications and Ethics (CHRPE) of the School of Medicine and Dentistry at the Kwame Nkrumah University of Science and Technology, Ghana, reviewed and approved the study protocol (CHRPE/AP/231/18). Written informed consent was obtained from all participants. For participants below 18 years and incapacitated patients (those with altered level of consciousness), consent was sought from their parents or designated guardians. The study was conducted in accordance with the principles guiding the conduct of research in human subjects as set out in the Declaration of Helsinki [18].

## Results

A total of seventy (78) participants were recruited. One patient whose signs and symptoms of neurological disease was confirmed to be cerebral malaria was excluded from the study. Seventy-seven (77) patients were enrolled unto the study from the Paediatric (48, 62.3%), Emergency and Internal Medicine (29, 37.7%) units of KATH. The participants were from 27 towns and cities in eight (8) of the 16 administrative regions of Ghana with the Ashanti Region being represented by 80.5% (62) of the study participants. The age of the patients ranged from 4 months to 68 years with a mean age of 13.8 years (median age = 5.0 years, interquartile range (IQR) = 14.5 years). Most participants (47, 61.0%) were less than 10 years and only 3 participants (3.9%) were above 60 years. More males (50, 64.9%) than females were included in the study. Christians represented 76.6% of the study participants and the least represented religion was traditional (2, 2.6%) as presented in Table 2.

**Table 2:** Demographic characteristics of study participants.

Variable	Frequency	Positive viral PCR test (≥ 1 virus), n (%)
**Age (years)**		
<10	47 (61.0)	18 (38.3)
10–19	14 (18.2)	7 (50.0)
20–29	3(3.9)	3 (100.0)
30–39	3(3.9)	0 (0.0)
40–49	3(3.9)	1 (33.3)
50–59	4 (5.2)	0 (0.0)
≥60	3(3.9)	2 (66.7)
**Gender**		
Male	50 (64.9)	17 (34.0)
Female	27 (35.1)	14 (51.9)
**Religion**		
Islam	14 (18.2)	5 (35.7)
Christianity	59 (76.6)	24 (40.7)
Traditional	2(2.6)	1 (50.0)
Unknown	2 (2.6)	1 (50.0)
**Level of education**		
Preschool	27 (35.1)	10 (37.0)
Basic	35 (45.5)	15 (42.9)
SHS/Vocational	10 (13.0)	4 (40.0)
Tertiary	3 (3.9)	1 (33.3)
No formal education	2 (2.6)	1 (50.0)

### Clinical presentation

The participants frequently presented with fever (66, 85.7%), seizures (62, 80.5%), lethargy (50, 64.9%) and headache (39, 50.6%). Other clinical presentations included restlessness (27, 35.1%), neck stiffness (27, 35.1%), neck pain (20, 26.0%), jaundice (13, 16.9%), cognitive dysfunction (14, 18.2%) and head trauma (9, 11.7%). Most of the patient were conscious and alert (34, 44.7%) with reduced level of consciousness seen in 27 participants (35.5%). Fifteen of the participants (19.7%) were unconscious. Table 3 shows the clinical characteristics of the study participants and the number (percentage) of patients who were positive for at least one virus under the respective symptoms.

**Table 3:** Clinical characteristics of study participants.

Symptom	Frequency (%)	Positive viral PCR test (≥ 1 virus), n (%)
**Fever**	66 (85.7)	29 (43.9)
**Seizure**	62 (80.5)	26 (42.0)
**Lethargy**	50 (64.9)	20 (40.0)
**Vomiting**	47 (61.0)	20 (42.6)
**Headache**	39 (50.6)	16 (41.0)
**Diarrhoea**	34 (44.2)	16 (47.1)
**Neck stiffness**	27 (35.1)	10 (37.0)
**Restlessness**	27 (35.1)	10 (37.0)
**Neck pain**	20 (26.0)	6 (30.0)
**Cognitive dysfunction**	14 (18.2)	5 (35.7)
**Jaundice**	13 (16.9)	7 (53.8)
**Head trauma**	9 (11.7)	1 (11.1)
**Hydrophobia**	4 (5.2)	3 (75.0)
**State of consciousness**		
Conscious	34 (44.7)	13 (38.2)
Somnolence	27 (35.5)	11 (40.7)
Unconscious	15 (19.7)	7 (46.7)

### Viral aetiology

PCR results were generated and delivered to clinicians for patient management in approximately 6 hours after sample collection. Viruses were detected in 31 (40.3%) of the study participants in either CSF, rectal or oral swab samples. Viral aetiology was confirmed (CSF positive) for 19 participants (24.7%) and 12 participants (15.6%) had a probable encephalitis [positive in oral (7) or rectal swab (5) only). PCR testing (of CSF, rectal and oral specimens) was negative in 46 (59.7%) of the participants. Mono-infections (23, 74.2%) as well as coinfections (8, 25.8%) were detected. The coinfections consisted of enterovirus/cytomegalovirus (3, 37.5%), herpes simplex/cytomegalovirus (4, 50.0%), herpes simplex/cytomegalovirus/enterovirus/rabies virus (1, 12.5%). The most frequently detected virus in all samples was CMV (15, 48.4%), enteroviruses (12, 38.7%) and HSV (9, 29.0%). Rabies (4, 12.9%) and VZV (1, 3.2%) were also detected (Table 4). Cytomegalovirus was detected in CSF of 6 patients (31.6%). Other viruses that were detected in CSF as well as coinfections detected in CSF are as detailed in Table 5.

**Table 4:** Aetiology, clinical outcomes, and management characteristics of patients.

Aetiology	Frequency	Outcome		Case fatality ratio
		Dead	Full recovery	
**Herpes simplex viruses**	9	4	5	0.44
**Varicella zoster virus**	1	1	0	1.00
**Human cytomegalovirus**	15	7	8	0.47
**Enteroviruses**	12	2	10	0.17
**Rabies virus**	4	4	0	1.00
**Antibiotic administered.**				
Yes	24	7	17	
No	7	6	1	
**Antiviral administered.**				
Yes	10	5	5	
No	21	8	13	

**Table 5:** Distribution of viruses among the CSF positive patients.

Virus	Frequency (%)	Outcome (Death)
	N = 19	N = 11
**Cytomegalovirus (CMV)**	6 (31.6)	3 (27.3)
**Enterovirus (ENTV)**	5 (26.3)	1 (9.1)
**Rabies virus (RABV)**	3 (15.8)	3 (27.3)
**Herpes simplex virus (HSV)**	1 (5.3)	1 (9.1)
**Varicella zoster virus (VZV)**	1 (5.3)	1 (9.1)
**CMV/ENTV**	1 (5.3)	0 (0.0)
**CMV/HSV**	1 (5.3)	1 (9.1)
**CMV/HSV/ENTV/RABV**	1 (5.3)	1 (9.1)

### Antibiotic and antiviral administration

Antibiotics were administered to 61 (79.2%) of the participants. 33 patients (54.1%) were given only one type of antibiotic, while 26 (42.6%) and 2 (3.3%) of the patients were on two and three different types of antibiotics respectively. Ceftriaxone was the most frequently administered antibiotic (52, 85.2%). Twenty-three patients (37.7%) received a combination of ceftriaxone and other antibiotics. The most frequently combined antibiotics were ceftriaxone and cefpodoxime (5, 21.7%). Acyclovir was administered to 10 (32.3%) of the patients who tested positive for at least a virus.

### Clinical outcome

All 77 recruited patients were hospitalised for clinical evaluation and management. While on admission, 21 of the patients (21/77, 27.3%) died. The average length of hospital stay was 10.6 days (SD = 8.0, range = 1–66 days). All four patients with rabies encephalitis died on the day of their admission. Information on deaths associated with other viruses are presented in Table 5. All deaths occurred within the first 30 days following admission. Among the deceased, 11 (52.7%) had a reduced Glasgow coma score (<15). The univariate logistic regression model identified age, gender, clinical presentations like vomiting and diarrhoea, and a negative PCR test for a virus as significant factors associated with death, however, in a multivariate analysis, only gender and a negative PCR test remained statistically significant predictors of death. While several factors, including educational status, headache, seizures, lethargy, altered mental status upon admission, and antibiotic administration, were independently associated with an increased risk of death (2–6 times), these factors were not statistically significant contributors to mortality. Patients who received antiviral treatment after confirming viral aetiology were found to have a 21.8% increased chance of survival, this observation was not statistically significant (p = 0.69). While the relationship between diarrhoea and mortality did not reach statistical significance in the multivariate analysis (p = 0.36), the odds ratio (OR) of 2.143 indicates that patients with diarrhoea were twice as likely to die compared to those without diarrhoea (Table 6).

**Table 6:** Univariate and multivariate analysis of factors associated with death.

Variable	Frequency	Dead (%)	Univariate analysis		Multivariate analysis	
			OR (95% CI)	P-value	OR (95% CI)	P-value
**Age (< 10yrs)**	47	8 (17.0)	3.728 (1.306–10.641)	0.01	0.343(0.081–1.461)	0.15
**Gender (Males)**	27	12 (44.4)	3.644 (1.279–10.586)	0.02	5.702(1.536–1.172)	0.01
**Education status (preschool)**	27	3 (11.1)	3.556 (0.662–19.108)	0.14		
**Seizures**	62	15 (24.2)	2.089 (0.638–6.834)	0.22		
**Lethargy**	50	11 (22.0)	2.086 (0.746–5.833)	0.16		
**Headache**	39	15 (42.9)	6.429 (6.672–61.469)	0.11		
**Diarrhoea**	34	5 (14.7)	3.437 (1.107–10.669)	0.03	2.143(0.424–0.833)	0.36
**Vomiting**	47	9 (14.1)	2.814 (1.005–7.887)	0.05	0.897 (0.166–4.838)	0.9
**Cognitive dysfunction**	14	2 (14.3)	2.591 (0.528–12.714)	0.24		
**Antibiotic administration**	61	14 (23.0)	2.611 (0.823–8.280)	0.10		
**Antiviral administration**	16	5 (31.3)	0.782 (0.235–2.600)	0.69		
**Viral aetiology**	31	13 (41.9)	0.291 (0.103–0.828)	0.02	0.178 (0.048–0.664)	0.01

### Risk factors associated with mortality

Risk factors associated with mortality included age, gender, and a negative PCR result. Mortality increased with increasing age, and patients below 10 years had a 65.7% chance of survival (OR = 0.343(0.081–1.461), *p = 0*.*15*). Females were about 6 times (OR = 5.702(1.536–1.172), *p = 0*.*01*) more likely to die from viral encephalitis compared to males, and patients with confirmed viral aetiology were more likely (82.2% chance) to survive (OR = 0.178(0.048–0.664), *p = 0*.*01*). Antibiotic administration proved detrimental among suspected viral encephalitis patients and increased the likelihood of death by a factor of 2.6 times in patients who were administered any form of antibiotic, however, it was not a statistically significant contributor to mortality (Table 6).

## Discussion

Incorporating laboratory diagnostics into clinical management reduces the burden on the patient as well as the healthcare services. Certain forms of encephalitis have specific treatments. In the case of the Herpesviruses, there are well-established antiviral treatments. Vaccination against rabies and supportive treatment strategies for antibody-mediated encephalitis also exist [19, 20]. The timely identification of aetiology is critical because these clinical neurological infections are life-threatening and therefore require rapid and life-saving laboratory tests to be performed for diagnosing the aetiological agents. Once aetiologies are established, therapeutic interventions become less challenging.

In our study, encephalitis predominantly occurred in young children, and a progressive reduction in incidence was observed with increasing age. This pattern was also observed in Brazil [21], Australia [22], Sweden [23], and England [24]. The male to female sex ratio (M/F) was 1.85:1. Although more males were recruited, the proportion of females that were positive for at least one virus (51.9%) was higher than that of males (34.0%), and females were more likely to die (*p = 0*.*01*). Mortality among the females seemed to increase with increasing age, suggesting that, older females are more likely to die of suspected viral encephalitis. This observation has not been described elsewhere and contrasts the findings of several studies [25–27] that seem to suggest that males are more susceptible to viral encephalitis and usually experience a bad outcome. The underlying cause for this observation remains unclear; however, it aligns with the findings of a study conducted in Senegal [28] suggesting that racial and geographic disparities could contribute to the observed differences between studies conducted in Africa and those conducted elsewhere. The frequent administration of antibiotics could also partly explain the observed disparity. In our study, although the administration of antibiotic to suspected encephalitis patients was not determined to be a statistically significant risk factor, the risk of death was 2.6 times higher in those who received any form of antibiotics. Majority of the female patients (18, 66.7%) received antibiotics and a poor outcome (death) was observed in 7 (38.9%).

Out of the 77 patients evaluated, 31 (40.3%) were found to have viral encephalitis, with an overall mortality of 27.3%. Herpes and enteroviruses were frequently identified as cause of encephalitis (Table 4). The prevalence of herpes viruses is likely a result of a complex interplay between viral and host immune factors. After initial exposure, the virus goes into a lifelong latency and remains dormant in the host until reactivation after evading host defenses. The replication activity of the viruses within the brain can damage the brain cells, leading to encephalitis [29]. The frequent association of enteroviruses with viral encephalitis agrees with those of other authors [30–32]. The viruses were mainly associated with encephalitis in children less than 10 years, which agrees with a study in children in the United States of America [33]. Diarrhoea (8/12) and vomiting (11/12) were frequent symptoms in individuals with enterovirus related encephalitis.

Patients who had a confirmed viral aetiology had an 82.2% chance of a better outcome (OR = 0.178 (0.048–0.664), *p = 0*.*01*). This observation is of clinical significance since confirmed aetiology translates directly into timely and more effective clinical management. Once viral aetiology is confirmed, antiviral therapy can begin. Acyclovir is effective against enteroviruses and those of the Herpesviridae family. From our study, patients who were put on antiviral therapy had a 21.8% chance of a better outcome (OR = 0.782 (0.235–2.600), *p = 0*.*69*). Similar findings were made in a Canadian study which estimated a 20% reduction in mortality for patients who received oral acyclovir [34].

The Association of British Neurologists, the British Infection Association and the Infectious Diseases Society of America recommend that patients with suspected encephalitis undergo CSF analysis to determine the underlying cause and receive prompt, and appropriate treatment [34]. Prompt diagnosis of viral aetiology of encephalitis and initiation of specific and supportive treatment strategies have been shown to improve patient outcomes [35]. Furthermore, studies have consistently demonstrated that early administration of acyclovir during the early stages of viral encephalitis, before the patient loses consciousness, is most effective in reducing both morbidity and mortality [36, 37]. There is also evidence to suggest that mortality of herpes simplex encephalitis is reduced Additionally, evidence suggests that early diagnosis and acyclovir treatment can reduce mortality rates of herpes simplex encephalitis by 10–20% [38, 39].

In our study, we observed that patients who were administered antiviral after confirmed viral aetiology had increased chances of survival. Also, the inability to confirm viral aetiology was significantly associated with death.

Overall, our results validate the crucial role of rapid diagnosis of viral aetiologies in viral encephalitis. Prompt identification of viral cause of encephalitis is critical for timely and effective clinical management, and confirmed viral aetiology leads to better outcomes. Antiviral therapy administered to patients with confirmed viral aetiology significantly improved their chances of a favourable outcome. This study also identified specific predictors of mortality in viral encephalitis, providing comprehensive insights into the disease in Ghana. The data obtained from our study also allows a better understanding of the epidemiologic and clinical profile of Encephalitis in Ghana. Incorporating the findings of our study into existing policies on the clinical management of encephalitis in Ghana has the potential to enhance the diagnosis and management of viral encephalitis cases, ultimately leading to better patient outcomes.

This study has some limitations. This was a single centre study; therefore, the generalisability of our findings might be limited. However, KATH receives admissions from the other 15 regions of Ghana. This makes it likely that the cases included are largely representative of the situation in the country. The study included a higher proportion of children than adults, potentially limiting the generalisability of the findings to the adult population. Additionally, the PCR panel only included six viruses, raising the possibility that some viral aetiologies may have remained undiagnosed. Future studies should employ expanded PCR panels to comprehensively identify a wider range of potential viral causes of encephalitis.

## Conclusion

Despite the limitations, this is the first study to comprehensively assess the viral aetiologies of encephalitis in Ghana and provides important evidence to aid health system planning and policy making. We show that molecular tools are vital for rapid diagnosis of viral encephalitis and aid clinicians to initiate early antiviral therapy to improve patient outcomes.

## Supporting information

S1 Checklist *PLOS ONE* clinical studies checklist. (DOCX)

S2 Checklist STROBE statement—checklist of items that should be included in reports of *cross-sectional studies*. (DOC)
